# Foveated glasses-free 3D display with ultrawide field of view via a large-scale 2D-metagrating complex

**DOI:** 10.1038/s41377-021-00651-1

**Published:** 2021-10-12

**Authors:** Jianyu Hua, Erkai Hua, Fengbin Zhou, Jiacheng Shi, Chinhua Wang, Huigao Duan, Yueqiang Hu, Wen Qiao, Linsen Chen

**Affiliations:** 1 School of Optoelectronic Science and Engineering & Collaborative Innovation Center of Suzhou Nano Science and Technology, Soochow University, 215006 Suzhou, China; 2 Key Lab of Advanced Optical Manufacturing Technologies of Jiangsu Province & Key Lab of Modern Optical Technologies of Education Ministry of China, Soochow University, 215006 Suzhou, China; 3 State Key Laboratory of Advanced Design and Manufacturing for Vehicle Body, College of Mechanical and Vehicle Engineering, Hunan University, 410082 Changsha, China; 4 SVG Optronics, Co., Ltd, 215026 Suzhou, China

**Keywords:** Displays, Lithography, Metamaterials

## Abstract

Glasses-free three-dimensional (3D) displays are one of the game-changing technologies that will redefine the display industry in portable electronic devices. However, because of the limited resolution in state-of-the-art display panels, current 3D displays suffer from a critical trade-off among the spatial resolution, angular resolution, and viewing angle. Inspired by the so-called spatially variant resolution imaging found in vertebrate eyes, we propose 3D display with spatially variant information density. Stereoscopic experiences with smooth motion parallax are maintained at the central view, while the viewing angle is enlarged at the periphery view. It is enabled by a large-scale 2D-metagrating complex to manipulate dot/linear/rectangular hybrid shaped views. Furthermore, a video rate full-color 3D display with an unprecedented 160° horizontal viewing angle is demonstrated. With thin and light form factors, the proposed 3D system can be integrated with off-the-shelf purchased flat panels, making it promising for applications in portable electronics.

## Abstract (web summary)

A space-variant information density three-dimensional display with ultrawide field of view is achieved by a large-scale 2D-metagrating complex, which shows great potential for portable electronic devices.

## Graphical abstract

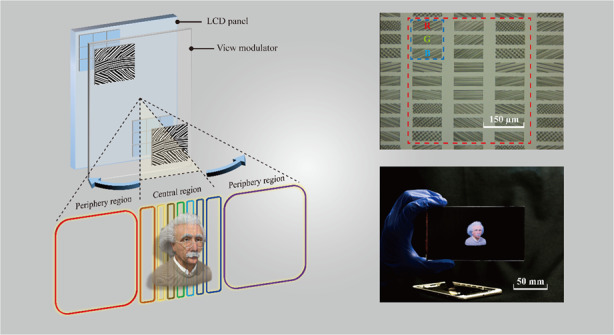

## Introduction

Glasses-free three-dimensional (3D) displays are regarded as one of the most promising technologies that will redefine portable electronics^1–7^, yet little progress has been made in the physical foundation of 3D displays. Multiview 3D displays discretize the light field into spaced views to provide a 3D stereoscopic experience for many observers simultaneously^8–16^. To obtain ideal 3D display utility, high-resolution 3D images should be projected to spaced views with smooth motion parallax over a large viewing angle^17,18^. However, limited by state-of-the-art display information, the spatial resolution, angular resolution, and viewing angle have become critical trade-offs in 3D displays^1,19^. For example, to build a reasonably good 3D display with a 1 K spatial resolution and a 3° angular resolution along the vertical and horizontal directions, a display panel with a 50 K resolution is required for a viewing angle of 150°, which is an order of magnitude greater than that of commercial products.

One solution is to exploit the temporal redundancy to increase the amount of display information^20–26^. For example, a multi-projection time-multiplexed 3D display has been proposed by using a steering screen to improve the angular resolution^23^. Most recently, a slim-panel holographic video display with an angular steering backlight has been introduced to increase the effective space-bandwidth product at the expense of a reduced refresh rate^26^. On the other hand, nature provides us with another strategy for addressing the critical trade-off among the spatial resolution, angular resolution, and viewing angle when display information is limited^27–30^. Recently, a foveated vision strategy was introduced in a single-pixel computational imaging system, thereby increasing the frame rate by reducing the number of pixels in the peripheral region^27^. In a vertebrate eye, the spatially variant visual density provides a high angular resolution of 1 min of arc at the central region of the retina (the fovea centralis), as well as a wide viewing angle of 160° without significantly increasing the total information that needs to be processed.

Inspired by the vertebrate eyes, we propose a general approach of 3D display, through which spatially variant information is projected based on the frequency of observation. Densely packaged views are arranged at the center, while sparsely arranged views are distributed at the periphery. In fact, package views in a gradient density are straightforward, but nontrivial. First, the angular separation of the views needs to be varied. Second, the irradiance pattern of each view has to be tailored so as to eliminate overlap between views to avoid crosstalk. Third, one should avoid gaps between views to ensure smooth transition within the field of view (FOV). As a result, views with hybrid dots, lines, or rectangle distributions are desirable to achieve gradient density. However, 3D display based on geometric optics, such as lenticular lens, microlens arrays, or pinhole arrays, can neither manipulate gradient view distribution nor expand the FOV^31–33^. The progress in planar diffractive optics based on photonics opened an opportunity for precise light field manipulation^34–37^. For example, a silicon-based metasurface diffractive optical element was reported for structured light projection over 120° FOV^38^. The full-color and dynamic 3D holography using metasurfaces made of subwavelength nanostructures with spatially varying orientations or sizes were also reported^39–42^. Metamaterials can provide superior light manipulation for 3D display, but the design and fabrication of nanostructures over a large scale for display (5–100 inch) application is a big challenge^43,44^.

To manipulate view distribution over a large scale, we design and propose a feasible strategy based on the two-dimensional (2D)-metagrating complex (2DMC). The 2DMCs are proposed to individually control both the propagation direction and the irradiance distribution of the emergent light from each 2D metagrating. As a result, the 3D display system provides a high spatial and angular resolution at the central viewing zone, i.e., the most comfortable observing region. Since the periphery viewing zone is less used in most occasions, we suppress the redundant depth information and broaden the FOV to a range comparable to that of a 2D display panel. Furthermore, a homemade flexible interference lithography (IL) system is developed to enable the fabrication of the view modulator with >1,000,000 2D metagratings over a size >9 inch. With total display information <4 K, a static or video rate full-color 3D display with an unprecedented FOV of 160° is demonstrated. The proposed 3D display system has a thin form factor for potential applications in portable electronic devices.

## Results

### The foveated glasses-free 3D display

**Fig. 1:**
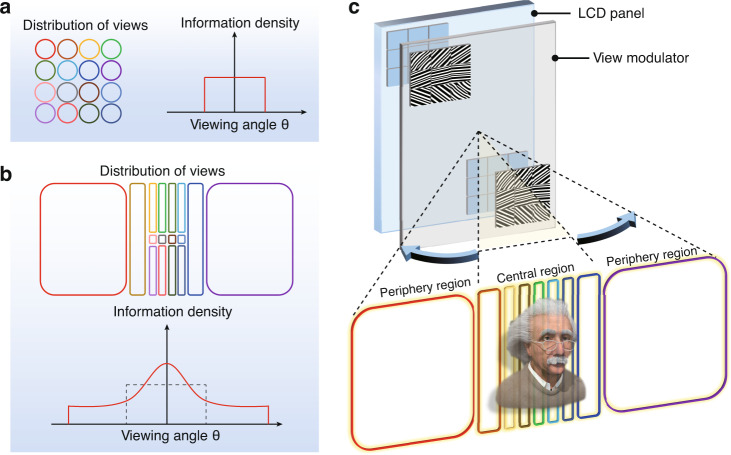
Working principle of the foveated glasses-free 3D display. **a** State-of-the-art glasses-free 3D display with uniformly distributed information. The irradiance distribution pattern of each view is a dot or a line for current 3D displays based on microlens or cylindrical lens array. **b** The proposed glasses-free 3D display with variant distributed information. The irradiance distribution pattern of each view consists of dots, lines, or rectangles. To make a fair comparison, the number of views (16 views) is consistent with **a**. **c** Schematic of a foveated glasses-free 3D display. An LCD panel matches the view modulator pixel by pixel. For convenience, two voxels are shown on the view modulator. Each voxel contains 3 × 3 pixelated 2D metagratings to generate View 1–View 9

Generally, the spatial resolution (multiview display pixels *N*_mul_) and the angular resolution (angular separation ∆*θ*) determine the visual experience provided by a multiview 3D display^3^. Therefore, we adopt the information density (pixels per degree (PPD)) to evaluate the performance of the 3D display:

1 $${\rm{ID}}= \frac{{N_{\mathrm{mul}}}}{{\Delta}\theta}$$

where ID represents the information density. A higher information density provides a higher spatial resolution with more fluidic motion parallax. In prior studies, constant information density was provided within the viewing angle by views with the same distribution pattern (Fig. 1a). In contrast, we propose 3D display with spatially variant information density by precisely manipulating the view distribution into hybrid dot/line/rectangle shape (Fig. 1b).

Figure 1c illustrates the schematic of the view modulator with 2DMCs. To generate a horizontally variant display information density, we define 9 irradiance patterns with variant widths. Pixelated 2D metagratings (3 × 3), which are grouped into a voxel, are designed to provide the predefined view distribution. We reserve detailed calculation of the 2D metagratings in the view modulator pixel by pixel to the Supplementary Information (Section 1). As a result, the information density distribution will be modulated as in the foveated vision.

### Design and fabrication of 2D metagratings

The cornerstone of the proposed display architecture is a large-scale 2DMC on the view modulator. With a size up to 9 inch, the data volume of 2DMCs is >1.8 Tb. Due to the large data volume, both the design and fabrication of 2DMCs is nontrivial.

**Fig. 2:**
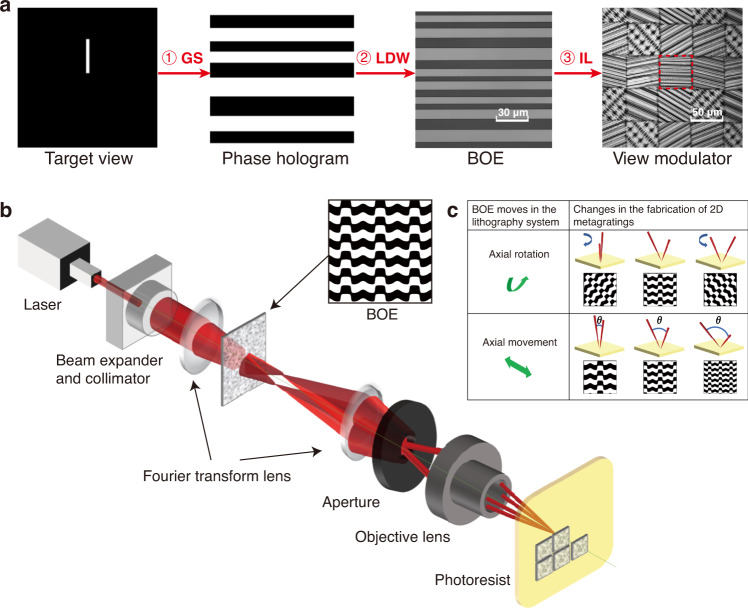
Fabrication process flow of 2DMCs on the view modulator. **a** Design and fabrication process for 2D metagratings in the view modulator: ① generating the phase hologram by the GS algorithm according to the target view distribution; ② fabricating the binary optical element (BOE) by a laser direct writing (LDW) system; ③ patterning 2DMCs on the view modulator by a self-developed interference lithography (IL) system. The red dashed line marks a pixelated 2D metagrating. **b** Schematic of the self-developed interference lithography system. **c** Illustration of controlling the BOE to adjust the scaling factor of periods and orientation of the patterned 2D metagratings

Figure 2a illustrates the schematic of the design process of 2DMCs. We first designed the phase hologram of the nanostructures according to the target view distribution by the Gerchberg–Saxton algorithm^45^. Four typical phase holograms responsible for typical view distributions are summarized in Table 1, and they can be applied in different scenarios. For example, dot-shaped views provide the highest information density. The vertically oriented and horizontally oriented line-shaped views reduce the information density in one direction while maintaining the information density in the other direction. The rectangular-shaped views reduce the information density along both directions, which are typically adopted for peripheral observing region. Although the diffractive pattern for each voxel is the same, the position of each pixel and the diffraction angle for the emergent beam varied. As a result, a unique nanostructure is donated to each pixel over the entire view modulator. Furthermore, with negligible tolerance, it has been proven that the 2D metagratings corresponding to the same view have similar shapes but with different scaling factors of periods and orientations (for additional information, see Section 2 in the Supplementary Information).

**Table 1:**
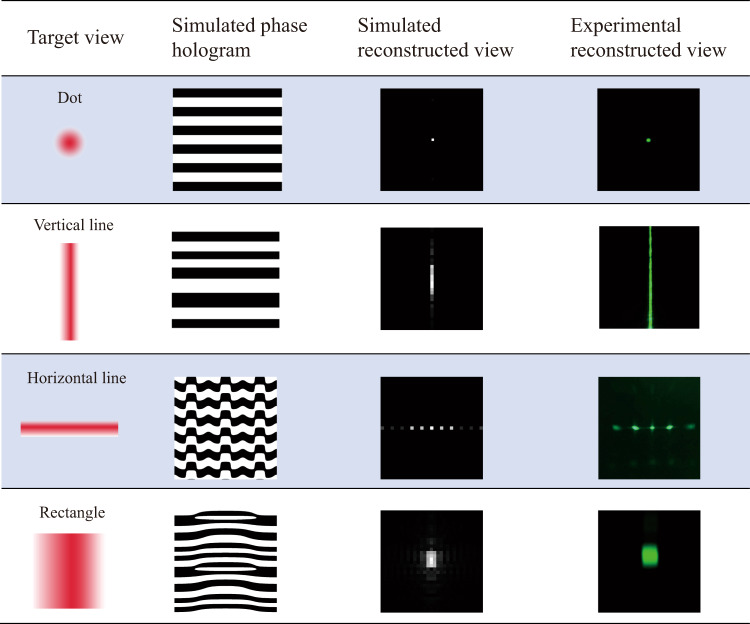
A summary of four typical phase holograms designed for various target views^a^

^a^For convenience, we maintained the first-order diffraction light and blocked out the other orders of light because only the first-order diffraction components were used

The fabrication of a view modulator with complex nanostructures remains a challenge. On the one hand, electron-beam lithography (EBL) is a typical nanopatterning tool in the laboratory^46–49^, but it suffers from limited throughput and size. On the other hand, laser direct writing (LDW) technology can fabricate patterns over several inches^50^, yet the minimum resolvable line width is diffraction limited to a submicron scale. Herein we developed a versatile IL system, as shown in Fig. 2b. A collimated and expanded laser beam illuminates a phase-modulated system, which consists of two Fourier transform lenses and a binary optical element (BOE) inserted in between. Then an interference pattern is formed by the multiple diffractive beams of the BOE at the back focal plane of the second Fourier transform lens. Finally, the interference pattern light field is minified by an objective lens and projected on the photoresist. The patterned structures on the photoresist are a minified multibeam interference pattern of the BOE. Details about the principles of our versatile IL system can be found in the Supplementary Information (Sections 2 and 3). Therefore, we enabled the fabrication of 2DMCs on the view modulator to form dot, linear, and rectangular hybrid shaped view distribution shown in Table 1.

Furthermore, the axial movement and axial rotation of the BOEs between two Fourier transform lenses lead to variations in the scaling factor of periods and orientation of the patterned 2D metagratings^51^, respectively (Fig. 2c). A pixelated 2D metagrating can be fabricated by pulse exposure. On the one hand, the 2DMCs for one view can be patterned by precisely controlling the scaling factor of periods and the orientation. On the other hand, the 2DMCs for views with different irradiance shapes can be achieved by inserting the corresponding BOEs in the homemade IL system. Furthermore, it is worth noting that the periodic tuning accuracy of fabricated 2D-metagrating can reach within 1 nm. The processing efficiency of the IL system can reach 20 mm^2^ mins^−1^, 500 times faster than the speed of EBL.

### Performance of the space-variant information density 3D display

**Fig. 3:**
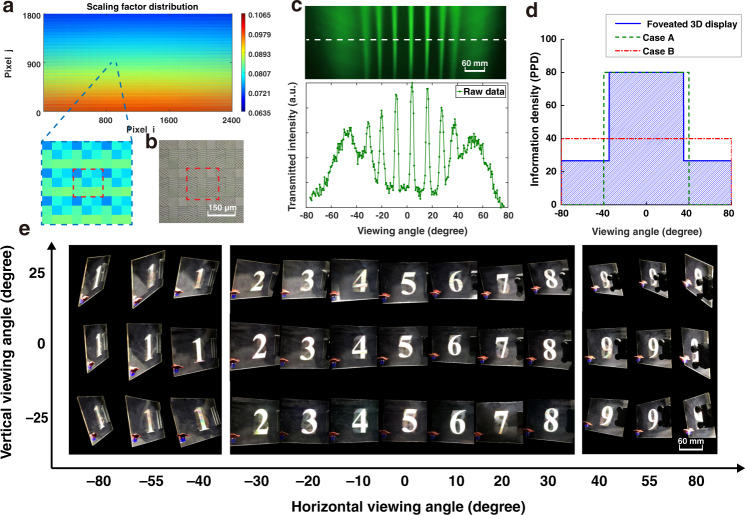
A proof of concept with horizontally variant information density. **a** Variation of the scaling factor for periods of the 2D metagratings. The blue dashed line marks an area containing 3 × 3 voxels. The red dashed line marks a voxel. **b** The microscopic image of the 2DMCs, captured by a laser confocal microscope (OLYMPUS, OLS4100). The red dashed line also marks a voxel. **c** The irradiance of view distribution and the intensity distribution along the white dashed line of the views. **d** The variant information density distribution (blue solid line) and its comparison with two cases for uniformly distributed information. Case A is that the angular separation between views is set to 10° with decreased FOV (green dashed line). In case B, the FOV is kept to 160°, but the information density is greatly reduced (red dashed line). **e** Images of numbers “1–9” observed from left to right views. A dinosaur toy is adhered to the left corner of the view modulator and is served as a reference for the viewing angle. See another 3D images in Figs. S5 and S7

Enabled by the homemade IL system, we fabricated several view modulators with different screen sizes and complexity (for details about the fabrication, see “Materials and methods”). The typical parameters of the three prototypes are summarized in Table 2. To prove the concept, we made a 6-inch view modulator with horizontal-variant information density. Figure 3a shows the variation of the scaling factor for periods of the 2D metagratings on the view modulator. The proposed 6-inch view modulator contains a total of 800 × 600 voxels, and each voxel is composed of 3 × 3 pixels for 9 views. That is to say, a total of 4,320,000 2D metagratings need to be patterned on the view modulator. A microscopic image of the 2DMCs on the view modulator is shown in Fig. 3b. Figures 3c and S4 presents the measured and simulated radiation pattern of the 9-view modulator prototype (for details about the simulation, see “Materials and methods”), respectively. Seven views (Views 2–8) are uniformly distributed in the central region with an angular separation of 10°, while the peripheral views (Views 1 and 9) cover 40° on each side of the central views. The crosstalk is measured as 14.88% (for detailed measurement, see Section 4 in the Supplementary Information). Compared with the theoretical value of 8%, a slight increment in experimental value is observed. Besides, the diffraction efficiency of 2DMC for red/green/blue (R/G/B) color is measured as 8.89, 7.72, and 11.92%, respectively. In contrast, the theoretical diffraction efficiency of 500 nm deep 2DMC is 20%. The experimental deviations for both crosstalk and diffraction efficiency are induced by nanofabrication inaccuracy and the misalignment in system assembly.

**Table 2:** Critical parameters of three typical prototypes

3D imaging characteristics	View modulator in Fig. 3e	View modulator in Fig. 5a	View modulator in Fig. 5b
Screen size	12 cm × 9 cm	5.4 cm × 5.4 cm	20.6 cm × 12.9 cm
View number	9	36	9
Field of view	160°	160°	160°
Number of pixels	2400 × 1800	3600 × 1200	1280 × 800
Sub-pixel size	50 μm × 50 μm	15 μm × 45 μm	108 μm × 108 μm
Angular separation	10° (center)	3° (center)	10° (center)
	30° (edge)	8.5° (edge)	30° (edge)
Information density^a^	80 PPD (center)	200 PPD (center)	42.6 PPD (center)
	26.7 PPD (edge)	70.6 PPD (edge)	14.2 PPD (edge)

^a^PPD: pixels per degree

A shadow mask with hybrid images of numbers is adopted to match the 9-view modulator pixel by pixel. When the light from a collimated light-emitting diode (LED) illuminates the prototype, we record the “1–9” numbers projected to each view, as shown in Fig. 3e. The horizontal FOV is 160°, and the vertical FOV is 50° (Visualization 1). The information density is modulated to 80 PPD at the central region and 26.7 PPD at the periphery (Fig. 3d).

**Fig. 4:**
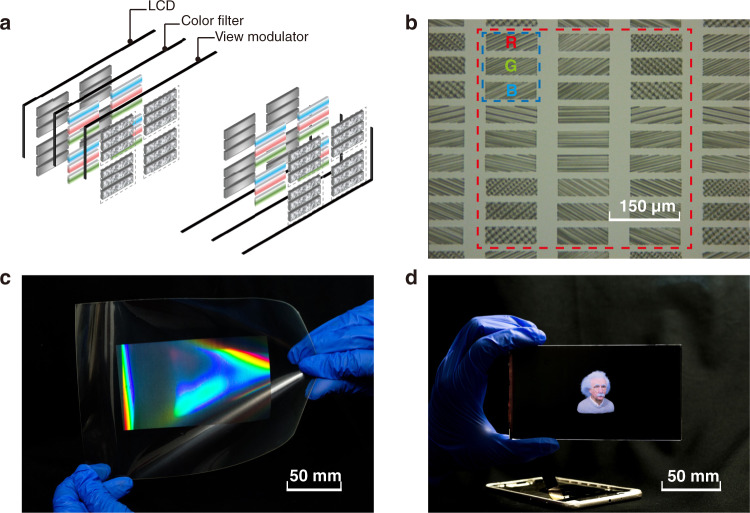
A full-color and video rate spatially variant information density 3D display. **a** Schematic of the full-color video rate 3D displays that contain an LCD panel, a color filter, and a view modulator. **b** The microscopic image of the RGB 2DMCs on the view modulator. The red dashed line marks a voxel containing 3 × 3 full-color pixels, and the blue dashed line marks a full-color pixel containing three subpixels for R (650 nm), G (530 nm), and B (450 nm). **c** Photo of the nanoimprinted flexible view modulator with a thickness of 200 µm. **d** A full-color, video rate prototype of the proposed 3D display. The backlight, battery, and driving circuit are extracted

For video rate full-color 3D displays, we successively stack a liquid crystal display (LCD) panel, color filter, and view modulator together to keep the system thin and compatible (Fig. 4a). Since most LCD panels have already been integrated with a color filter, the system integration can be simply achieved by pixel to pixel alignment of the 2D-metagrating film with the LCD panel via one-step bonding assembly. The layout of 2DMCs on the view modulator is designed according to the off-the-shelf purchased LCD panel (P9, HUAWEI) (Fig. 4b). To minimize the thickness of the prototype, 2D metagratings are nanoimprinted on a flexible polyethylene terephthalate (PET) film with a thickness of 200 µm (Fig. 4c), resulting in a total thickness of <2 mm for the whole system (Fig. 4d).

**Fig. 5:**
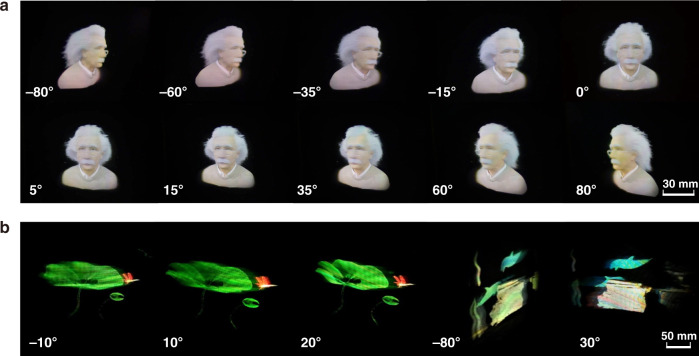
Performance of the foveated 3D display. **a** Images of “Albert Einstein” and **b** “whales” and “lotus leaves” observed from various views with natural motion parallax and color mixing. The number shown in the lower left corner represents the viewing angle of the image. See other 3D images in Fig. S6

A white LED light illuminates the 2D metagratings from the back with a filtered wavelength and modulated intensity. The emergent beams from R/G/B 2DMCs are combined for full-color display (Fig. 5a, b, Visualization 2 and 3). The FOV reaches a record of 160°. The information density is modulated to 200 PPD at the central region and 70.6 PPD at the periphery.

## Discussion

Here we achieved full-color 3D display with significantly suppressed color dispersion by several ways. First, since 2D-metagratings are wavelength sensitive, the structure of 2DMC is designed pixel by pixel according to the wavelength. Second, from the system point of view, the introduction of color filter significantly filters out the influence of color dispersion. Third, we pre-calibrate the white balance of the prototype. The displayed images can be pre-processed to further reduce the color dispersion.

In the experiment, we changed the information density by varying the angular resolution. In another strategy, we can assign more pixels to the central views, thereby the spatial resolution is increased as in the foveated vision.

Facilitated by the rapid advancement of nano-optics, we presented a general design strategy of glasses-free 3D display from view modulation aspect. The view modulator for multiple view projection is no longer a simple conjugate relation between image and object. We proved that 2DMC can be designed to precisely tailor the view distribution for gradient view arrangement and enlarged viewing angle. The view modulator with 2DMCs can be further designed to eliminate crosstalk or increase viewing depth. Moreover, by combining views with a fan-shaped irradiance pattern, a tabletop 3D display system with variant information density can be realized.

In summary, we propose a facile and robust approach for spatially variant information density 3D display with a large-scale 2DMC served as a view modulator. A homemade flexible IL system is developed to enable the nanopatterning of view modulator with increased complexity for portal electronic devices. As a result, high angular resolution is preserved in the central region, while a wide viewing angle is maintained. The display information is arranged nonuniformly based on the observing habit of human beings. Hence, we demonstrate a full-color, video rate 3D display with a thin form factor. The viewing angle sets a record of 160° for the glasses-free 3D display.

The demonstrated spatially variant information density 3D display opens a new avenue for glasses-free 3D displays by tackling the critical trade-off among the spatial resolution, angular resolution, and viewing angle. We anticipate the ultrawide-FOV foveated 3D display to be used in commercial applications, such as consumer electronic devices.

## Materials and methods

### Sample fabrication

(1) For the inserted BOE: First, a 2.5-inch quartz plate was precleaned and spin coated with hexamethyldisilazane (DisChem, SurPass 3000) and positive photoresist (MicroChemicals, AZ® P4620) at a total thickness of 1 μm. Then the quartz plate was micropatterned with various binary phase holograms using a homemade LDW system (SVG Optronics, MiScan200). After photolithography, the phase holograms were developed in a NaOH solution and blown dry. Finally, the BOE structures were etched to a depth of 700 nm, and the minimum period was approximately 7.5 μm. The BOEs were finally inserted in the self-developed IL system for the fabrication of view modulator.

(2) For the view modulators: First, a glass substrate was cut and precleaned. The glass substrate was then coated with positive photoresist (RUIHONG Electronics Chemicals, RJZ-390) at a thickness of 1 μm. Then the 2DMCs were successively patterned by a self-developed IL system. The pixel size of each unit can be adjusted according to the aperture size. It took 27 h to prepare a 6-inch 2DMC with 2400 × 1800 pixels. After IL, the nanopatterns were developed in a NaOH solution and blown dry, and then it was electroplated with a layer of nickel (NI) to make a master mold. The NI-plated mold was then used to imprint the 2D metagratings onto the polyurethane (PUA) resin, adopting roll-to-plate nanoimprint lithography. The PUA resin was then subsequently cured by ultraviolet light for 3 min. Finally, those 2DMCs were effectively mass transferred on a flexible PET membrane to form a view modulator.

### Numerical simulations

3D simulations were performed using the finite-difference time-domain (FDTD) method, and FDTD simulations were conducted using Lumerical’s FDTD solver. The refractive index of the photoresist was set as 1.476. We used a plane wave source with an incident angle of 30°, and the wavelength was 540 nm. We used Bloch and perfectly matching layer boundary conditions for the transverse and longitudinal directions, respectively. The practical 2DMCs were replaced with spatial-multiplexing gratings with multiple periods. The periods ranged from 600 to 1400 nm. The mesh accuracy was chosen as a compromise among the accuracy, memory requirements, and simulation time.

## Supplementary information

Supplementary information

Static 3D images from a 6-inch view modulator

A full color spatially variant information density 3D display

A video rate foveated 3D display
