# Supplementary material for: Foveated glasses-free 3D display with ultrawide field of view via a large-scale 2D-metagrating complex
Source: Light Sci Appl. 2021 Oct 12;10:213. doi: 10.1038/s41377-021-00651-1 (PMC8511001; doi:10.1038/s41377-021-00651-1)
Supplement: Supplementary file 1 — Supplementary information [file 41377_2021_651_MOESM1_ESM.docx]

Supplementary Information for

**Foveated glasses-free 3D display with ultrawide field of view via a large-scale 2D-metagrating complex**

Jianyu Hua^1,2^†, Erkai Hua^1,2^†, Fengbin Zhou^1,2^, Jiacheng Shi^1,2^, Chinhua Wang^1,2^, Huigao Duan^3^, Yueqiang Hu^3^, Wen Qiao^1,2^*, Linsen Chen^1,2,4^*

^1^School of Optoelectronic Science and Engineering & Collaborative Innovation Center of Suzhou Nano Science and Technology, Soochow University, Suzhou 215006, China

^2^Key Lab of Advanced Optical Manufacturing Technologies of Jiangsu Province & Key Lab of Modern Optical Technologies of Education Ministry of China, Soochow University, Suzhou 215006, China

^3^State Key Laboratory of Advanced Design and Manufacturing for Vehicle Body, College of Mechanical and Vehicle Engineering, Hunan University, Changsha 410082, China

^4^SVG Optronics, Co., Ltd, Suzhou 215026, China

**Corresponding author. Email:* [*wqiao@suda.edu.cn*](mailto:wqiao@suda.edu.cn)*;* [*lschen@suda.edu.cn*](mailto:lschen@suda.edu.cn)

†*These authors contributed equally: Jianyu Hua and Erkai Hua*

This document provides supplementary information to “Foveated glasses-free 3D display with ultrawide field of view via a large-scale 2D-metagrating complex”.

Section 1. Light field modulation by the large-scale 2D-metagrating complex

According to the Raman-Nath regime^1,2^, the vectors of the 2D-metagrating complex (2DMC) can be calculated by the relationship between the incident beam and diffraction beam (see Fig. S1):

$k_{dm}=k_{i}-G_{m}\text{ }$ (S1)

where $\left| G_{m} \right|=2\pi/\Lambda_{m}$ are the vectors of the 2DMCs; $\Lambda_{m}$ are a set of periods in the pixelated 2D metagratings; $m$ is the period serial number in each pixel $(m=1,2,3\cdots)$; and $\left| k_{i} \right|=2n\pi/\lambda$ and $\left| k_{dm} \right|=2\pi/\lambda$ are the vectors of incident beam and diffraction beam, respectively. The periods in each pixel can be calculated as:

$\frac{1}{\text{Λ}_{mx}}=\frac{n\cos\alpha_{1}-\cos\alpha_{m2}}{\lambda}\text{ }$ (S2)

$\frac{1}{\text{Λ}_{\text{my}}}=\frac{n\cos\beta_{1}-\cos\beta_{m2}}{\lambda}\text{ }$ (S3)

where $\lambda$ is the beam wavelength; $n$ is the refractive index of the photoresist; $\Lambda_{mx}$ and $\Lambda_{my}$ are the components of the 2D-metagrating periods along the x-axis and y-axis, respectively; $\alpha_{1}$ and $\beta_{1}$ are the components of the incident angle along the x-axis and y-axis, respectively; and $\alpha_{m2}$ and $\beta_{m2}$ are the components of the diffraction angle along the x-axis and y-axis, respectively. Combining Eq. (S2) and Eq. (S3), the multiple periods can be written as:

$\begin{matrix} \text{Λ}_{m}=\sqrt{\frac{\lambda^{2}}{(n\cos\alpha_{1}-\cos\alpha_{m2})^{2}+(n\cos\beta_{1}-\cos\beta_{m2})^{2}}}\text{ } \\ \text{ =}\sqrt{\frac{[(x_{0m}-x)^{2}+(y_{0}-y)^{2}+z_{0}^{2}]\lambda^{2}}{(x_{0m}-x)^{2}+(n\sin\theta\sqrt{(x_{0m}-x)^{2}+(y_{0}-y)^{2}+z_{0}^{2}}-y_{0}+y)^{2}}} \end{matrix}$ (S4)

The multiple orientation angles in each pixel from the y-axis can be written as:

 (S5)

where $\theta$ is the incident angle from the z-axis; x and y are the coordinates of the nanostructure pixel at the view modulator; and $x_{0m}$, $y_{0}$ and $z_{0}$ are the coordinates of the radiation pattern of views.

Therefore, the projection position of the radiation-pattern views is determined by the incident beam and the nanostructure vectors. In other words, we can calculate the 2D metagratings in the view modulator pixel by pixel according to the views.

Section 2. Operating principles of the versatile interference lithography system

Fig. S2 illustrates the schematic of the proposed versatile IL system. We assume that the optical transmittance of the binary optical element (BOE) can be written as $t\left( x_{0},y_{0} \right).$ The light field at the focal plane of the first Fourier transform (FT) lens is:

$\begin{matrix} U(x,y)=c'\exp[jk\frac{x^{2}+y^{2}}{2(f_{1}-d)}]\times\int_{-\infty}^{+\infty} \int_{-\infty}^{+\infty} t(x_{0},y_{0})\exp(-jk\frac{xx_{0}+yy_{0}}{f_{1}-d})dx_{0}dy_{0} \\ \text{ }=c'\exp[jk\frac{x^{2}+y^{2}}{2(f_{1}-d)}]\times T[\frac{x}{(f_{1}-d)\lambda},\frac{y}{(f_{1}-d)\lambda}] \end{matrix}$(S6)

where $x_{0}$ and $y_{0}$ are the coordinates of the BOE plane; $x$ and $y$ are the coordinates of the focal plane of the first FT lens; $d$ is the distance between the first FT lens and the BOE; $f_{1}$ and $f_{2}$ are the focal lengths of the first FT lens and the second FT lens, respectively; and the light field at the focal plane of the second FT lens is:

$\begin{matrix} U(x_{1},y_{1})=c'c''\int_{-\infty}^{+\infty} \int_{-\infty}^{+\infty} U(x,y)\exp(-jk\frac{xx_{1}+yy_{1}}{f_{2}})dxdy \\ \text{ }=c'''\times t[-\frac{f_{1}-d}{f_{2}}x_{1},-\frac{f_{1}-d}{f_{2}}y_{1}] \end{matrix}$ (S7)

where $c^{'},$ $c^{''}, c'''$ are constants and $x_{1}$ and $y_{1}$ are the coordinates of the focal plane of the second FT lens. According to the Fourier transform theorem, the light field distribution at the focal plane of the second FT lens is similar to the phase of BOE (see Table 1). In other words, the 2DMCs patterned by the same BOE (thereby generating the same pattern view) have similar shape but with different scaling factors of periods and orientation.

Take one typical BOE for a vertical-oriented line shaped view as an example. The optical transmittance can be written as:

$t(x_{0},y_{0})=\sum_{\text{m}} \cos(2\pi a_{m}x_{0})$ (S8)

where $a_{m}$ are the multiple spatial frequencies of the inserted BOE; $m$ is the serial number of spatial frequencies in each pixel $(m=1,2,3\cdots)$; From the aforementioned analysis, the modulated light field at the focal plane of the second FT lens can be written as:

$U(x_{1},y_{1})=\sum_{\text{m}} A\cos[j2\pi\frac{a_{m}(f_{1}-d)}{f_{2}}x_{1}]$ (S9)

Finally, the minified interference pattern formed by the objective lens on the photoresist can be written as:

$U(x_{2},y_{2})=\sum_{\text{m}} A\cos[j2\pi\frac{a_{m}M(f_{1}-d)}{f_{2}}x_{2}]$ (S10)

Based on this principle, the spatial frequencies of the 2D metagrating patterned on the photoresist are:

$F_{\text{m}}=\frac{a_{m}M(f_{1}-d)}{f_{2}}$ (S11)

The multiple periods of the fabricated 2D metagrating in a pixel can be written as:

$\overline{\text{Λ}_{m}}\text{=}\frac{\text{Λ}_{0m}f_{2}}{M(f_{1}-d)}\text{ }$ (S12)

where $\Lambda_{0m}$ are the multiple periods of the inserted BOE; $m$ is the period serial number in each pixel $(m=1,2,3\cdots)$; *M* is the demagnification of the objective lens; and *A* is a constant. Although the 2D metagrating in one pixel have various periods for an extended view, the scaling factor of periods *P* of which is the same:

$P\text{=}\frac{f_{2}}{M(f_{1}-d)}$ (S13)

Therefore, the multiple periods of the output 2DMCs can be controlled simultaneously by adjusting the BOE (thereby changing the scaling factor of periods). From the above, the self-developed IL system can manufacture nanostructure pixels with various scaling factors of periods and orientation according to calculation results.

Section 3. Analysis of experimental approximation

As shown in Fig. S1, the irradiance pattern from the 2DMCs that are responsible for the same view should be identical. However, due to the experimental approximation induced by interference lithography, the position of the views deviates from the calculation results, which are analyzed in this section.

To simplicity, we assume that each 2D metagrating in the view modulator produces a horizontally extended view with two points [see Fig. S8(a)]. According to Eq. (S4), the theoretical periods in a pixel are:

$\text{Λ}_{1}\text{=}\sqrt{\frac{[(x-x_{0})^{2}+y^{2}+z_{0}^{2}]\lambda^{2}}{(x-x_{0})^{2}+[n\sin\theta\sqrt{(x-x_{0})^{2}+y^{2}+z_{0}^{2}}+y]^{2}}}$ (S14)

$\text{Λ}_{2}\text{=}\sqrt{\frac{[(x-x_{0}+W)^{2}+y^{2}+z_{0}^{2}]\lambda^{2}}{(x-x_{0}+W)^{2}+[n\sin\theta\sqrt{(x-x_{0}+W)^{2}+y^{2}+z_{0}^{2}}+y]^{2}}}$ (S15)

According to Eq. (S12), the periods of fabricated 2D metagratings are:

$\overline{\text{Λ}_{1}}\text{=}\frac{\text{Λ}_{01}f_{2}}{M(f_{1}-d')}$ (S16)

$\overline{\text{Λ}_{2}}\text{=}\frac{\text{Λ}_{02}f_{2}}{M(f_{1}-d')}$ (S17)

where $d^{'}$ is the distance between the first Fourier transform lens and BOE in the interference lithography system. Combing Eq. (S14) and Eq. (S16), the scaling factor of periods in a pixel is:

$P=\frac{1}{\text{Λ}_{01}}\sqrt{\frac{[(x-x_{0})^{2}+y^{2}+z_{0}^{2}]\lambda^{2}}{(x-x_{0})^{2}+[n\sin\theta\sqrt{(x-x_{0})^{2}+y^{2}+z_{0}^{2}}+y]^{2}}}$ (S18)

Then, Eq. (S17) can be rewritten as:

$\overline{\text{Λ}_{2}}=\frac{\text{Λ}_{02}}{\text{Λ}_{01}}\sqrt{\frac{[(x-x_{0})^{2}+y^{2}+z_{0}^{2}]\lambda^{2}}{(x-x_{0})^{2}+[n\sin\theta\sqrt{(x-x_{0})^{2}+y^{2}+z_{0}^{2}}+y]^{2}}}$ (S19)

The experimental deviation in periods of 2D metagratings is:

$\Delta\text{Λ}_{2}=\sqrt{\frac{[(x-x_{0}+W)^{2}+y^{2}+z_{0}^{2}]\lambda^{2}}{(x-x_{0}+W)^{2}+[n\sin\theta\sqrt{(x-x_{0}+W)^{2}+y^{2}+z_{0}^{2}}+y]^{2}}}-\frac{\text{Λ}_{02}}{\text{Λ}_{01}}\sqrt{\frac{[(x-x_{0})^{2}+y^{2}+z_{0}^{2}]\lambda^{2}}{(x-x_{0})^{2}+[n\sin\theta\sqrt{(x-x_{0})^{2}+y^{2}+z_{0}^{2}}+y]^{2}}}$ (S20)

where $W$ is the horizontal width of the view; *x* and *y* are the coordinates of the nanostructure pixel at the view modulator; $x_{0}$ and $z_{0}$ are the coordinates of the first point in the view; and $\Lambda_{01}$ and $\Lambda_{02}$ are the periods in the inserted BOE. Fig. S9(a) shows the variation of the experimental deviation in periods $\Delta\Lambda_{2}$ on the view modulator. The maximum periodic deviation over the entire view modulator is less than 14 nm. As shown in Fig. S9(b), the relative experimental deviations in periods are approximately 1%. The mean deviation in the horizontally extended view result from the fabrication approximation is approximately 8.12%.

We also analyze the condition of vertical extension of views [see Fig. S8(b)]. The experimental deviation in periods of 2D metagratings is:

$\Delta\text{Λ}_{2}'=\sqrt{\frac{[x^{2}+(y-y_{0}+L)^{2}+z_{0}^{2}]\lambda^{2}}{x^{2}+[n\sin\theta\sqrt{x^{2}+(y-y_{0}+L)^{2}+z_{0}^{2}}-y_{0}+L+y]^{2}}}-\frac{\text{Λ}_{02}'}{\text{Λ}_{01}'}\sqrt{\frac{[x^{2}+(y-y_{0})^{2}+z_{0}^{2}]\lambda^{2}}{x^{2}+[n\sin\theta\sqrt{x^{2}+(y-y_{0})^{2}+z_{0}^{2}}-y_{0}+y]^{2}}}$ (S21)

where $L$ is the vertical length of the view; $y_{0}$ and $z_{0}$ are the coordinates of the first point in the view; and $\Lambda_{01}'$ and $\Lambda_{02}'$ are the periods in the inserted BOE. Fig. S9(c) shows the variation of the experimental deviation in periods $\Delta\Lambda_{2}'$ on the view modulator. The maximum periodic deviation is less than 15 nm. As shown in Fig. S9(d), the relative experimental deviations in periods are approximately 3%. The mean deviation in the vertically extended view due to the experimental approximation is approximately 29.54%.

One should note that the fabrication induced deviation of irradiance pattern and position for each view can be offset beforehand in the calculation. Thus, the home-made interference lithography system provides a versatile patterning tool for 2D metagratings used in 3D display.

Section 4. Details of the measurement

Crosstalk is determined by

$CT=\frac{I_{\text{all}}-I_{0}}{I_{0}}$ (S22)

where $I_{0}$ is the light intensity of one view when other views were blocked, and $I_{\mathrm{all}}$ is the light intensity of one view when all views were turned on. We measured $I_{0}$ and $I_{\mathrm{all}}$ successively by an optical power meter (PM400K1, Thorlabs). The crosstalk is measured as 14.88%.

The diffraction efficiency can be calculated by

$\eta=\frac{\sum I_{x,y}}{I_{i}}$ (S23)

where $I_{i}$ is the total light intensity impinged on the 2DMC, and $I_{x,y}$ is the diffraction light intensity at the position (*x,y*).

As shown in Fig. S10(a), we first measured the intensity of incident light $I_{i}$. A collimated LED light passed through an aperture and a reference plate. The reference plate is adopted to remove the influence of reflection. The transmitted light was focused on a light power meter, and measured as $I_{i}$. In the second step, we measured the light intensity of diffraction beam as shown in Fig. S10(b). The LED light passed through the same aperture and then diffracted by 2DMC on view modulator. A light power meter was adopted to measure the light intensity of diffracted beam at different position as $I_{x,y}.$ We calculate the total light intensity of diffraction beam $\sum I_{x,y}$, and corresponding diffraction efficiency of 2DMC for each wavelength. The diffraction efficiency for R/G/B color is 8.89%, 7.72% and 11.92%, [respectively](javascript:;).

References

1. Wan, W. Q. *et al*. Holographic sampling display based on metagratings. *iScience* **23**, 100773 (2020).
2. Harvey J. E. & Vernold, C. L. Description of diffraction grating behavior in direction cosine space. *Applied Optics* **37**, 8158–8159 (1998).


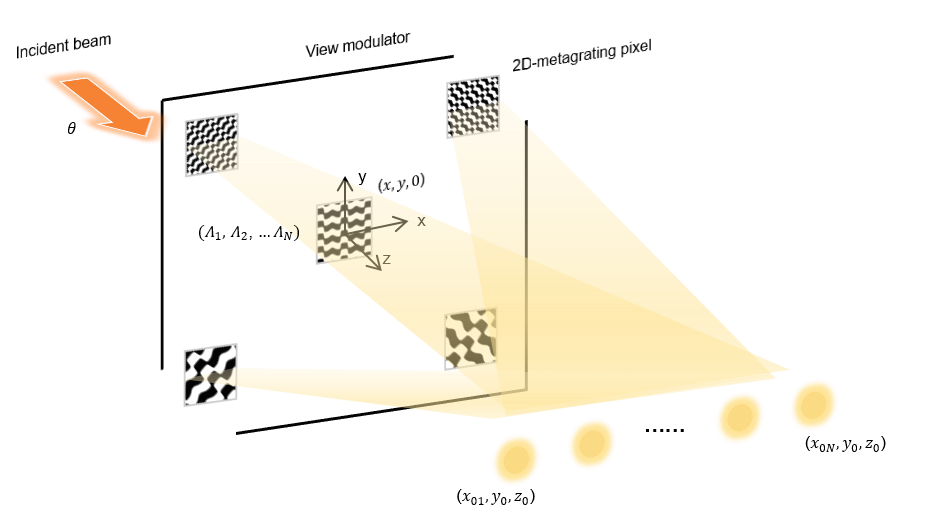


**Fig. S1.** Schematic of the light field modulation for one view by 2DMCs in the view modulator. $\Lambda_{1}, \Lambda_{2},\cdots\Lambda_{N}$ are a set of periods in one pixelated 2D metagrating. $x_{01}, x_{02}, \cdots x_{0N}$ are the coordinates along the x-axis in the extended view. Five pixels shown in the view modulator are responsible for the same view, which have a similar shape but with different orientation and periodic scaling factors.


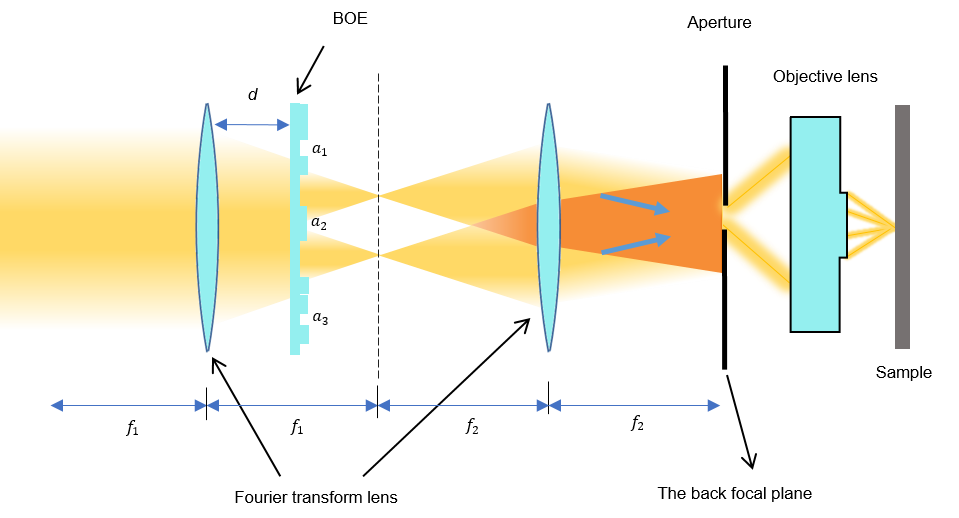


**Fig. S2.** Schematic of the homemade versatile interference lithography system. $f_{1}$ and $f_{2}$ represent the focal lengths of the two Fourier transform lenses. $d$ is the distance between the first lens and the binary optical element (BOE). $a_{1}$, $a_{2}$, and $a_{3}$ are the multiple spatial frequencies of the inserted BOE.


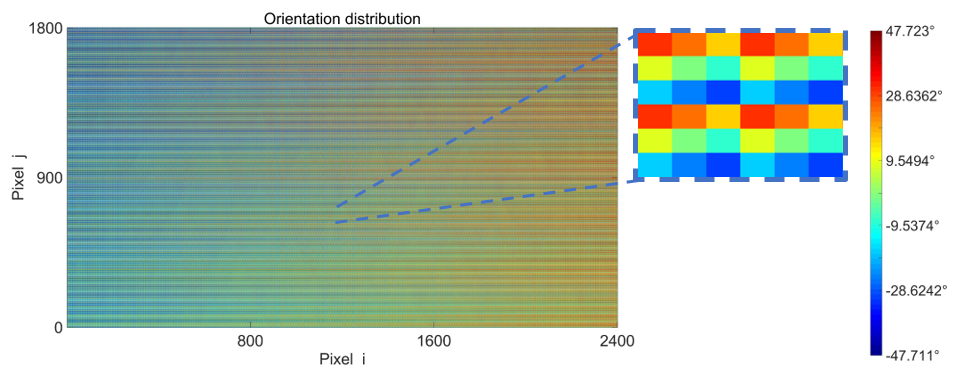


**Fig. S3.** Variation in the mean orientation of the 2DMCs on a 6-inch view modulator. The insert illustrates the variation of structural orientation in an area containing 2×2 voxels, and each voxel is composed of 3×3 pixels for 9 views. Related to Fig. 3a.


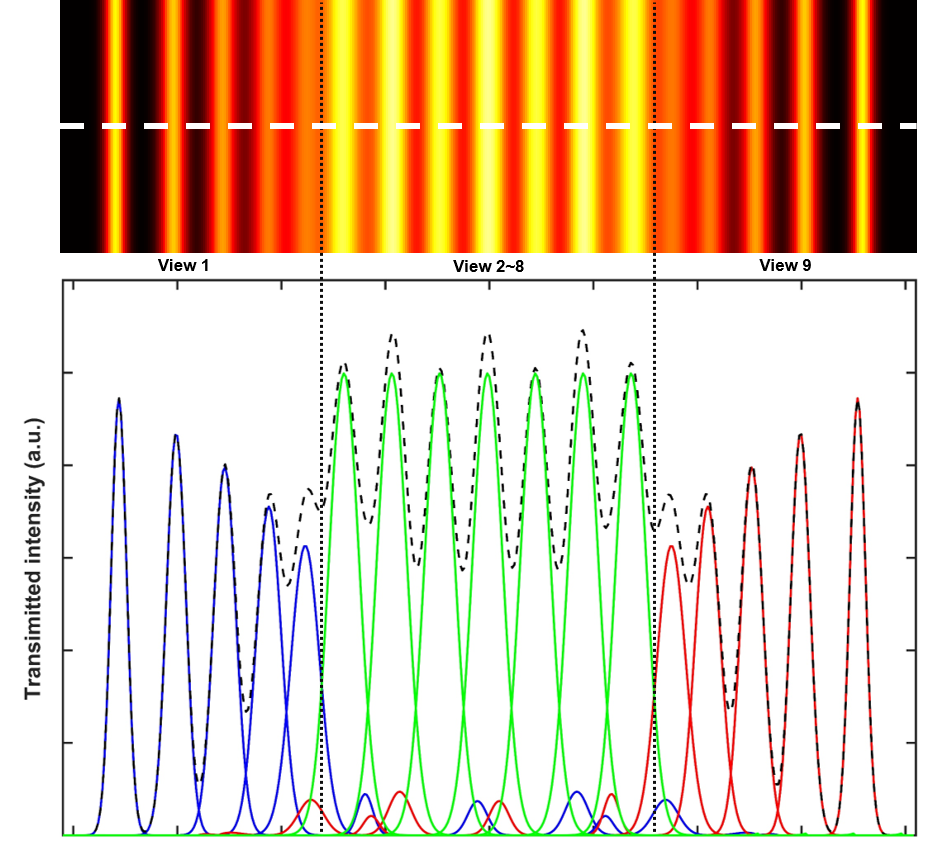


**Fig. S4.** FDTD simulation of the radiation pattern and intensity distribution along the white dashed line of the views. The diffraction efficiency is 20% for 500 nm deep 2DMC. The irradiance of high-order diffracted beams contributes to a crosstalk of around 8%.


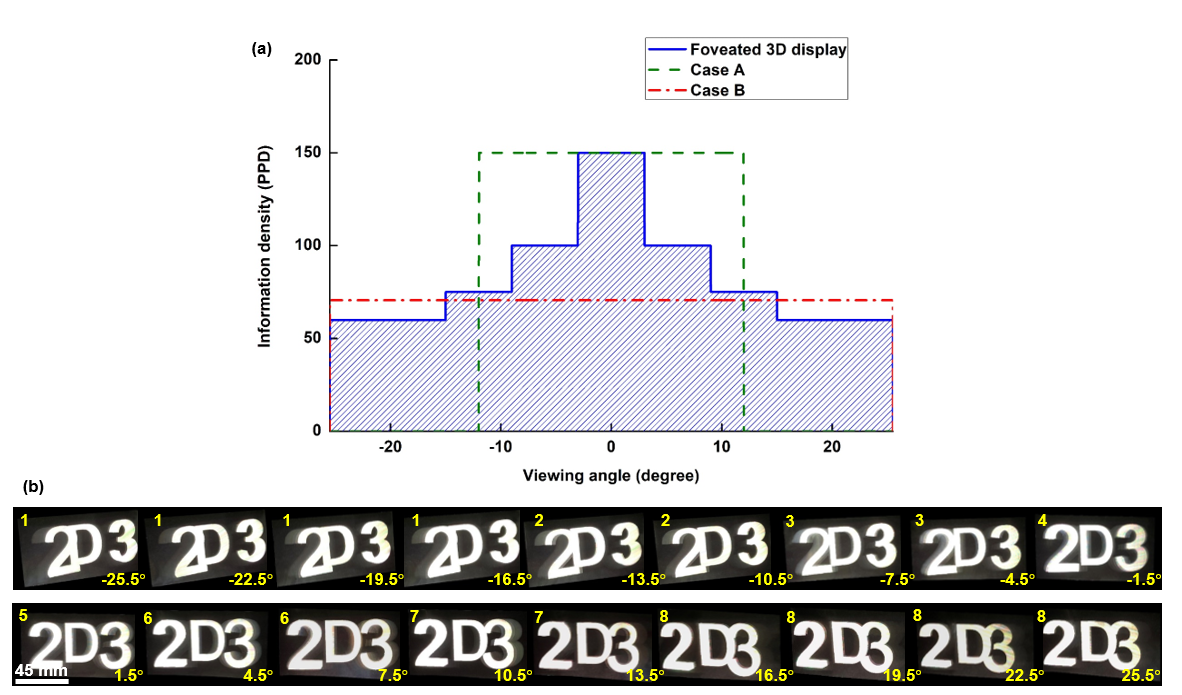


**Fig. S5.** Another prototype of the static 3D display with an FOV of 51°. (a) The variant information density distribution of the proposed system (blue solid line) and its comparison with two cases of uniformly distributed information. In case A, the angular separation between views is set to 3°, but the FOV is reduced to 24° (green dashed line). The FOV is kept to 51° (red dashed line) in case B, but the information density is decreased. (b) Images of the characters ‘2D3’ observed from various views. Related to Fig. 3e.


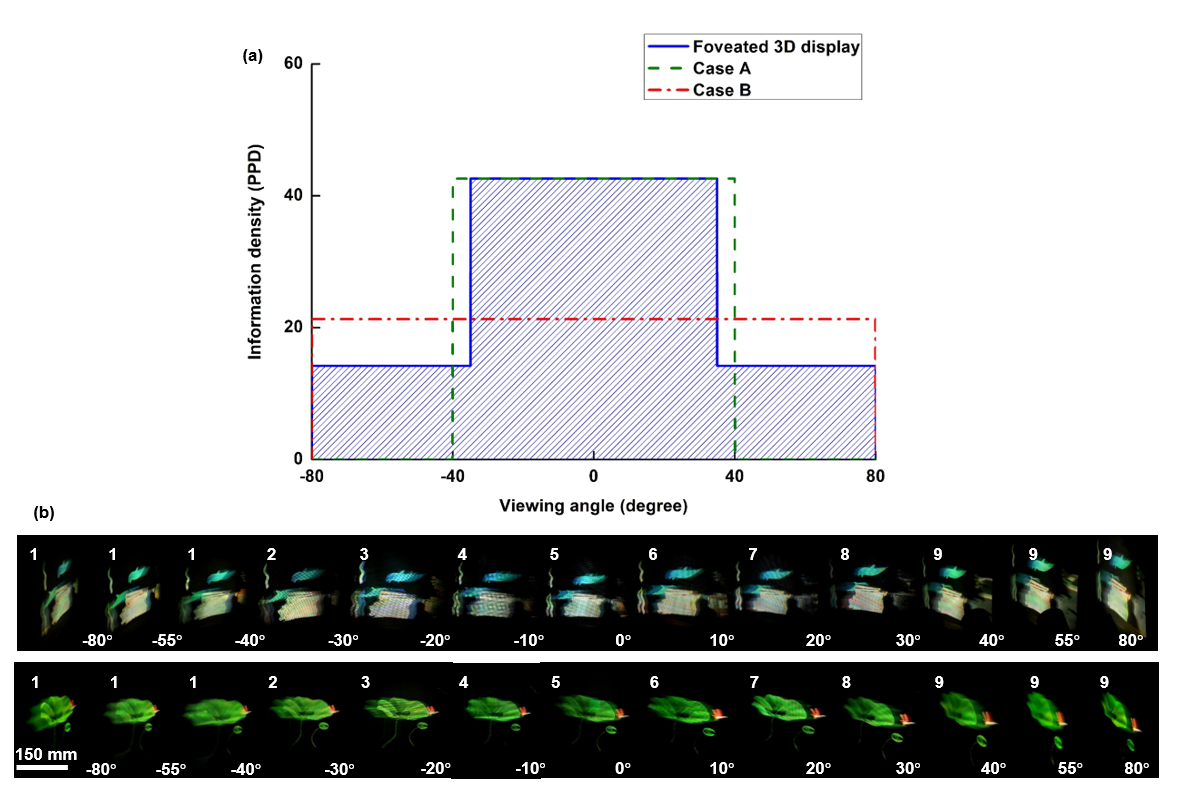


**Fig. S6.** A full-color, video rate 3D display prototype with an FOV of 160°. (a) The variant information density distribution of the proposed system (blue solid line) and its comparison with two cases of uniformly distributed information. In case A, the angular separation between views is set to 10°, but the FOV is reduced to 80° (green dashed line). The FOV is kept to 160° (red dashed line) in case B, but the information density is decreased. (b) the ‘whale’ and ‘lotus leaf’ images observed from various views. Related to Fig. 5b.


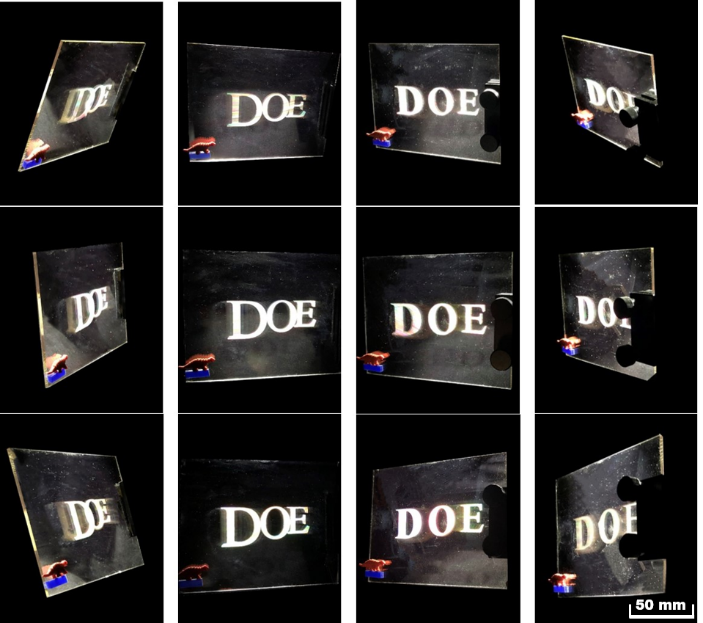


**Fig. S7.** A static 3D display prototype with an FOV of 160°. Images of the letters ‘DOE’ observed from left to right views showing an ultra large horizontal FOV of 160° and a large vertical FOV of 50°. A dinosaur toy is adhered to the left corner of the view modulator and is served as a reference for the viewing angle. Related to Fig. 3e.


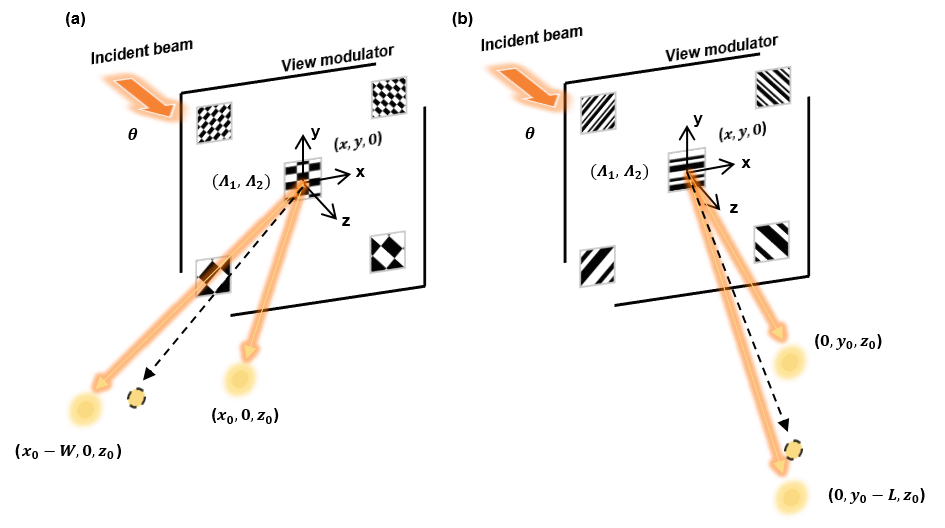


**Fig. S8.** The schematic of views deviation caused by the experimental approximation. (a) The deviation in the horizontally extended view. *W* is the horizontal width of the view. (b) The deviation in the vertically extended view. *L* is the vertical length of the view.


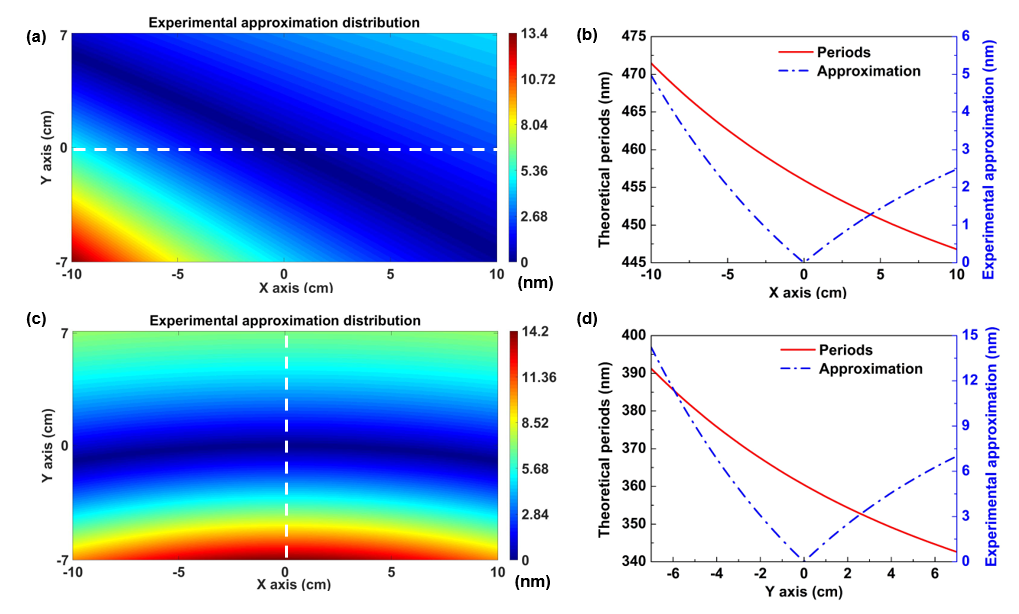


**Fig. S9.** Variation in the experimental approximation of 2DMCs on the view modulator. (a) Experimental approximation distribution in the horizontally extended view modulator. (b) Variation of experimental approximation and theoretical periods in the cross section along the white dashed line from Fig. S8(a). ($z_{0}$= 25 cm; *W* = 5 cm; $\lambda$= 530 nm; *n* = 1.5; $\theta$ = 30°; $\Lambda_{02}/{\Lambda_{01}}=$ 0.9856; $x_{0}=$ -$z_{0}\tan60\boldsymbol{^{\circ}}$ cm) (c) Experimental approximation distribution in the vertically extended view modulator. (d) Variation of experimental approximation and theoretical periods in the cross section along the white dashed line from Fig. S8(c). ($z_{0}$= 25 cm; *L* = 5 cm; $\lambda$= 530 nm; *n* = 1.5; $\theta$ = 30°; $\Lambda_{02}^{'}/{\Lambda_{01}'}=$ 0.9471; $y_{0}=$ -$z_{0}\tan40^{\circ}$ cm)


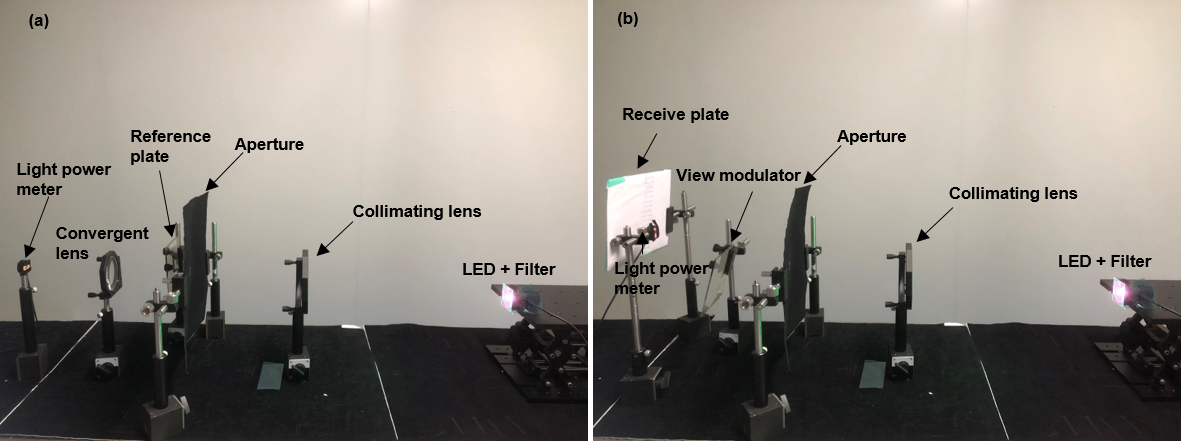


**Fig. S10.** Measurement of the diffraction efficiency with (a) the intensity of incident light $I_{i}$ and (b) the light intensity of diffracted beam at different position as $I_{x,y}$.

Supplementary Video Captions

**Visualization 1.** Static 3D images from a 6-inch view modulator. This video shows the ‘1-9’ numbers static 3D images projected to various views. Note the horizontal FOV is 160°, and the vertical FOV is 50°. It is corresponding to Fig. 3e in the main article.

**Visualization 2.** A full color spatially variant information density 3D display. This video shows the full color 3D images of 'Albert Einstein', created by integrating an off-the-shelf purchased LCD panel with a 200 µm-thick nanostructured membrane. Note the horizontal FOV is 160°. It is corresponding to Fig. 5a in the main article.

**Visualization 3.** A video rate foveated 3D display. This video shows a foveated 3D display with ‘two swimming whales’ (View 4 and View 6).
